# Fabrication of UV-Crosslinked Flexible Solid Polymer Electrolyte with PDMS for Li-Ion Batteries

**DOI:** 10.3390/polym13010015

**Published:** 2020-12-23

**Authors:** Sandugash Kalybekkyzy, Al-Farabi Kopzhassar, Memet Vezir Kahraman, Almagul Mentbayeva, Zhumabay Bakenov

**Affiliations:** 1 National Laboratory Astana, Department of Chemical and Materials Engineering, School of Engineering and Digital Sciences, Nazarbayev University, Nur-Sultan 010000, Kazakhstan; sandugash.kalybekkyzy@nu.edu.kz (S.K.); alfarabi.kopzhasar@gmail.com (A.-F.K.); 2 Institute of Batteries LLC., Nur-Sultan 010000, Kazakhstan; 3 Department of Chemistry, Marmara University, Istanbul 34722, Turkey; mvezir@marmara.edu.tr

**Keywords:** solid polymer electrolyte, polydimethylsiloxane, ion conductivity, lithium-ion battery, flexible battery

## Abstract

Conventional carbonate-based liquid electrolytes have safety issues related to their high flammability and easy leakage. Therefore, it is essential to develop alternative electrolytes for lithium-ion batteries (LIBs). As a potential candidate, solid-polymer electrolytes (SPEs) offer enhanced safety characteristics, while to be widely applied their performance still has to be improved. Here, we have prepared a series of UV-photocrosslinked flexible SPEs comprising poly(ethylene glycol) diacrylate (PEGDA), trimethylolpropane ethoxylate triacrylate (ETPTA), and lithium bis(trifluoromethane sulfonyl)imide (LiTFSI) salt, with the addition of polydimethylsiloxane with acrylated terminal groups (acryl-PDMS) to diminish the crystallinity of the poly(ethylene glycol) chain. Polysiloxanes have gained interest for the fabrication of SPEs due to their unique features, such as decrement of glass transition temperature (T_g_), and the ability to improve flexibility and facilitate lithium-ion transport. Freestanding, transparent SPEs with excellent flexibility and mechanical properties were achieved without any supporting backbone, despite the high content of lithium salt, which was enabled by their networked structure, the presence of polar functional groups, and their amorphous structure. The highest ionic conductivity for the developed cross-linked SPEs was 1.75 × 10^−6^ S cm^−1^ at room temperature and 1.07 × 10^−4^ S cm^−1^ at 80 °C. The SPEs demonstrated stable Li plating/stripping ability and excellent compatibility toward metallic lithium, and exhibited high electrochemical stability in a wide range of potentials, which enables application in high-voltage lithium-ion batteries.

## 1. Introduction

In recent years, the development of rechargeable lithium-ion batteries (LIBs) with enhanced electrochemical performance and safety has gained exceptional importance due to the growing need for sustainable energy and power supply, the miniaturization of devices, and the rapid expansion of the portable electronics market [1,2,3]. Particularly, flexible LIBs has become a field of intensive research, as a promising power source for forthcoming flexible and wearable electronics such as smart cloth, roll-up displays, bio-implants, foldable devices, and sensing smart displays [4,5]. Nevertheless, eco-compatibility, safety, and appropriate packaging remain a challenging task. The majority of the safety problems of batteries such as leakage, flammability, and toxicity are caused by liquid electrolytes [6]. Moreover, all components of such batteries are required to have sufficient flexibility, mechanical strength, and durability. Therefore, there is a necessity for the development of a new generation of safe all-solid-state flexible batteries with solvent-free electrolytes [7,8]. 

Solid polymer electrolytes (SPEs) are very promising for application in flexible batteries due to their advantages over inorganic electrolytes, like excellent flexibility, a wide range of electrochemical stability, high thermal and electrochemical stability, and ease of thin film formation [9]. Polymer-based electrolytes are prepared by dissolution of Li salts in a polar polymer matrix, where Li^+^ ions coordinate with the electron-donor groups of the polymer host. Ion conduction occurs by the electric field which forces ions to hop from one coordinating site to another [10,11]. Generally, polymers in such systems/membranes contain both crystalline and amorphous regions. Basically, ion transport occurs in the amorphous region of the polymer membrane [12]. Wright et al. reported the first study of ionic conductivity in crystalline poly(ethylene oxide) (PEO) with alkali iodide salts [13]. Later Armand applied PEO/alkaline salt complexes as electrolytes for lithium batteries [14]. Since that, a great deal of research has been done on the development of PEO based SPEs for all-solid-state batteries [15,16]. However, the application of PEO-based SPEs is limited due to their low ionic conductivity at ambient temperature (10^−8^–10^−7^ S cm^−1^), which is mainly related to the crystalline nature of PEO [17]. Additionally, PEO has a low dielectric constant, which restricts the fully dissociation of lithium salts, resulting in their agglomeration in solid polymer electrolytes. Furthermore, poor mechanical properties, resulting from their melting transition, may cause short circuits at high temperature [18]. As a result, various strategies have been applied to improve the ionic conductivity of PEO-based SPEs, such as structural modification, optimization of Li salt content, and the addition of organic plasticizer and inorganic fillers [19]. The introduction of an amorphous region into the polymer structure is an important approach, which can enhance the ionic conductivity of SPE, since the movement of polymer chains and flexibility greatly affects the ion transport [20]. It is widely known that comb-shaped SPEs with polyacrylates, polyphosphazenes, and polysiloxanes have a high flexibility and amorphous nature. Among them, polysiloxanes have both exceptional chemical, and physical, properties due to flexibility of the Si–O–Si bond and the low glass transition temperature (T_g_), which favor Li-ion conduction, as well as low toxicity and high chemical stability when used in solid electrolytes [21,22,23]. However, polysiloxane-based polymers are too soft at room temperature, therefore, the preparation of freestanding, dimensionally stable solid-state films requires additional modifications. Hydrosilylation, sol-gel reactions, curing process at higher temperatures, thiol-ene click reaction, and UV polymerization are the general approaches for preparation of polysiloxane-based ion conductive polymer systems [24]. There have been several works on polysiloxane containing SPEs, where they were combined with polyethers, PEO, plasticizers, and inorganic particles [18,25,26]. Nevertheless, in those systems ion conductivity improvement at ambient temperature led to poor dimensional stability and flexibility [27,28]. Crosslinking or adding an appropriate amount of conductive polymer to the structure are effective methods for improving the dimensional stability of SPEs [28,29]. Cross-linked polymers provide many advantages, including improved thermal stability, enhanced mechanical strength, and elimination of crystallinity. Among the various polymerization processes, UV-assisted radical polymerization (UV-photopolymerization) is an economical, fast, and nontoxic method, especially for networked polymer fabrication [30]. It is a room temperature curing process with rapid solidification of the polymer film, within several seconds or minutes, thus reducing overall energy and space consumption. UV-polymerization is very attractive for the development of safe and reliable solid-state electrolytes for LIBs, which is the main purpose of this study.

Therefore, in this paper we developed free-standing solid polymer electrolytes, using a one step and simple method, which facilitated the development of flexible and mechanically durable all-solid-state LIBs. The cross-linked SPEs were developed by UV-photopolymerization of ethylene glycol units containing oligomers and poly(dimethylsiloxane) (PDMS) with modified terminal groups. Composing PDMS into a crosslinked structure of poly(ethylene glycol) diacrylate (PEGDA) and trimethilolpropane ethoxylate triacrylate (ETPTA) decreases the crystallization of PEG and enhances the mechanical strength. The ethoxy groups of both PEGDA and ETPTA ensure the transport of Li^+^ ions within the polymer network [31]. Transparent SPEs with excellent flexibility and mechanical properties were achieved without any additional backbone, despite the high content of lithium salt, which was enabled by their networked structure, the presence of polar functional groups, and amorphous phase. The PDMS containing SPEs have improved ionic conductivity (~10^−6^ S cm^−1^), at lower temperature compared to conventional PEO based solid state electrolytes (10^−8^, ~10^−7^ S cm^−1^) [32], and demonstrate stable Li plating/stripping behavior and cycling stability toward metallic Li.

## 2. Materials and Methods 

Hydroxy-terminated poly(dimethylsiloxane) (PDMS, viscosity of 65 cSt), acryloyl chloride (≥97% purity), poly(ethylene glycol) diacrylate (PEGDA, M_n_ = 575), trimethilolpropane ethoxylate triacrylate (ETPTA, M_n_ ~428), trichloromethane (CHCl_3_, ≥99.5% purity), dichloromethane (CH_2_Cl_2_, anhydrous, ≥99.8% purity) tetrahydrofuran (THF, anhydrous, ≥99.9% purity), triethylamine (Et_3_N, ≥99.0% purity), K_2_CO_3_ (anhydrous, ≥99.0% purity), MgSO_4_ (anhydrous, 99.5% purity), 4-Dimethylaminopyridine (DMAP, ≥99%), 2-hydroxy-2-methylpropiophenone (HMPP, ≥97% purity), and lithium bis(trifluoromethane)sulfonimide (LiTFSI, 99.95%) were purchased from Sigma-Aldrich (Taufkirchen, Germany city, state country) and used as received. 

### 2.1. Acrylation Process of the PDMS 

The terminal groups of HO-(PDMS)_n_-OH were converted to photosensitive acrylate groups (OAcryl) to obtain AcrylO-(PDMS)_n_-OAcryl (a-PDMS) polymer, following the reported method [33]. HO-(PDMS)_n_-OH (16.5 g, (6 mmol)) and DMAP (0.00732 g, 0.065 mmol) were dissolved in CHCl_3_ (35 mL), placed in a 500 mL round bottom flask, and mechanically stirred by an overhead stirrer under a flow of N_2_ gas. After gradual addition of Et_3_N (1.214 g, 12 mmol) to the solution, acryloyl chloride (1.086 g, 12 mmol) was added dropwise over 30 min. The mixture was refluxed at 45 °C for 20 h with mild stirring. CHCl_3_ was removed by rotary evaporator, and the residual viscous product dissolved in ethyl acetate. Triethylamine hydrochloride salts were removed from the product by vacuum filtration, and ethyl acetate was removed under reduced pressure. The remaining product was dissolved in CH_2_Cl_2_ (~50 mL), and washed with 2 M K_2_CO_3_ solution (15 mL). The isolated viscous solution was dried with MgSO_4_, filtered out, and solvents were removed under reduced pressure.

### 2.2. Solid Electrolyte Fabrication

SPEs composed of a-PDMS, PEGDA, ETPTA, and Li salt (LiTFSI) were prepared by a UV-crosslinking method. In the first step, a-PDMS, PEGDA, and ETPTA (abbreviated as PPE) were homogeneously mixed in various stoichiometric mass ratios by stirring at room temperature. Separately, LiTFSI was dissolved in THF with the mass ratio of 2:1, respectively, and mixed with the PPE blend under continuous stirring for 30 min. The molar ratio of ethylene oxide unit to LiTFSI [EO]/[Li^+^] was varied to 0, 6, 18, and 30. Additionally, SPEs with different a-PDMS contents (0, 5, 10, and 15 wt %) were also investigated. The 2-hydroxy-2-methylpropiophenone (HMPP) was used as a photoinitiator, and its concentration was fixed as 3 wt % of solution. Then, the sample was placed between two glass plates to prepare the thin polymer film. The casted samples were exposed to UV-irradiation for 3 min by a UV lamp (Spectroline, ENF-240C/FE, λ_max_ = 365 nm). UV-crosslinked polymer films were easily removed from the glass plate, and placed into a heating chamber glovebox (MasterLab, MBraun, Germany) for 18 h at 60 °C under vacuum, in order to remove residual solvent. The overall process was performed in an argon-filled glove box (MasterLab, MBraun, Germany). The solvent residue and soluble contents of all polymer electrolyte systems were determined by calculating the gel fraction of the films without LiTFSI salt. The gel fraction of the samples was determined by weighing the samples before and after solvent extraction in acetone at room temperature for 24 h. Insoluble residue was dried in a vacuum oven at 60 °C overnight, and the gel content was calculated by the following equation:

[(W_2_)/W_1_] × 100% = Gel fraction, % (or cross-linked SPE) (1)

where W_1_ is weight of the SPE before washing with the solvent, in grams; W_2_ is weight of the SPE after washing with the solvent and drying in a vacuum oven at 60 °C [34].

### 2.3. Characterization and Measurements

The UV-crosslinked polymer films were investigated by Fourier transform infrared spectroscopy (FTIR-ATR, Nicolet iS10 FT-IR Spectrometer, Madison, WI USA) with a frequency range of 400−4000 cm^−1^. The gel fraction of the samples was determined by weighing the samples before and after solvent extraction in acetone at room temperature for 24 h. Insoluble residue was dried in a vacuum oven at 60 °C, and the gel content was calculated. The morphologies were characterized by field-emission scanning electron microscopy and energy-dispersive spectroscopy (FESEM, SEM/EDS, JEOL JSM-7500F, ZEISS Crossbeam 540, Jena, Germany). The T_g_ of the materials was determined by differential scanning calorimetry (DSC, Pyris Diamond, PerkinElmer, Shelton, CT, USA). The samples were cooled down to −100 °C, then heated at a rate of 10 °C min^−1^ up to 100 °C. The same conditions were used for all samples, and the heat flow values were collected from the second heating cycle. The thermal stability was tested by thermo-gravimetric analysis (TGA), using a simultaneous thermal analyzer (STA6000, Waltham, MA, USA) in a temperature range of 20–700 °C under nitrogen atmosphere, and with a heating rate of 10 °C min^−1^. The mechanical properties of the freestanding SPEs were analyzed by standard tensile stress–strain tests to measure modules, ultimate tensile strength, and elongation at break. The tensile measurements were performed at room temperature on a material testing machine Z010/TN2S (Kennesaw, GA, USA), using a crosshead/elongation speed of 1 mm/min.

The ionic conductivity of SPEs was determined by electrochemical impedance spectroscopy (EIS). The coin cells (CR2032, Hohsen Corp., Japan) were assembled with UV-crosslinked PPEs sandwiched between stainless steel (SS|PPE|SS). The impedance measurements were carried out from 10 Hz to 1 MHz with a voltage of 0.1 mV in amplitude, from room temperature to 80 °C, and with a temperature interval of 10 °C. The ionic conductivity was calculated by the following equation:

*σ* = *l*/(*R*_b_ × *r*^2^ × π) (2)

where *l* is the thickness, and *r* and *R*_b_ represent radius and bulk resistance of the crosslinked SPEs, respectively. *R*_b_ is the intercept on the real axis at the high frequency end of the Nyquist plot of complex impedance. The dependence of ionic conductivity and temperature was also fitted by the Vogel–Tamman–Fulcher (VTF) equation:

(3) $$\sigma =AT^{-1/2}exp\left[\frac{-B}{k_{b}\left(T-T_{0}\right)}\right]$$

In this equation, B is the activation energy related to the segmental motion of the polymer chains, $k_{b}$ is Boltzmann’s constant, $T_{0}$ is ideal glass transition temperature, which is generally taken as −50 °C from $T_{g}$, and A is a pre-exponential factor, which is also known as a frequency factor [35,36]. The electrochemical stability of the electrolyte was investigated in a CR2032 coin cell, with stainless steel as the working, and Li metal as the reference, electrode, respectively, and cross-linked PPE films as an electrolyte. Cyclic voltammetry (CV) and linear sweep voltammogram (LSV) tests were performed at 60 °C within a potential window of −1.5 V to 1.5 V vs. Li/Li^+^ at a scan rate of 1 mV s^−1^, and 1.5 V to 7.0 V at a scan rate of 0.1 mV s^−1^, respectively. The PPE SPE electrochemical stability was tested by assembling SS|SPE|SS blocking cells. Additionally, a symmetric cell test was performed at 60 °C, sandwiching SPE between two lithium metallic disks, which was charged/discharged for 30 min under a current density of 0.2 mA cm^−2^ for each step. All measurements were carried out using a VMP-3 potentiostat/galvanostat (Bio-Logic Instruments, France) within coin cells assembled in an argon-filled glove box (MasterLab, MBraun, Germany).

## 3. Results and Discussion

**Figure 1:**
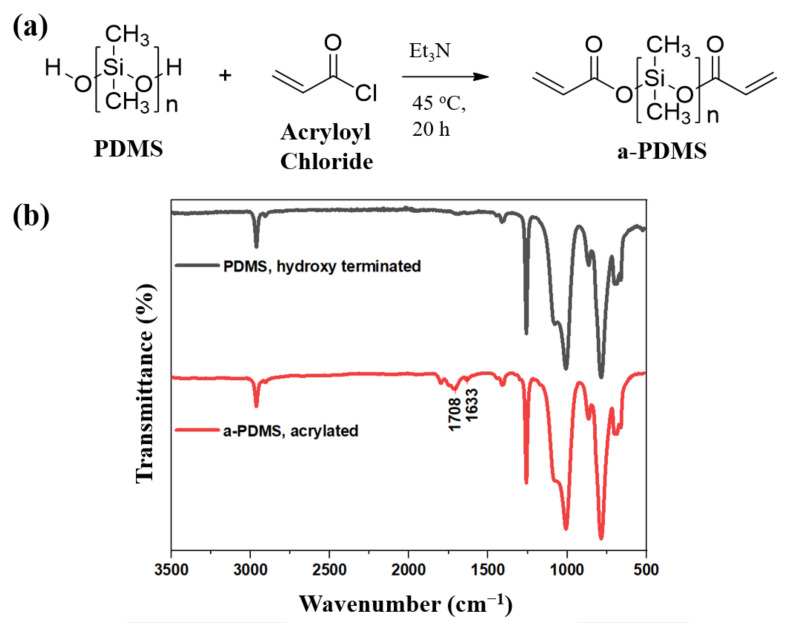
(**a**) Poly(dimethylsiloxane) (PDMS) acrylation reaction scheme, and (**b**) FTIR spectroscopy of PDMS and a-PDMS.

Prior to preparing PPE (a-PDMS, PEGDA and ETPTA) polymer films, photosensitive AcO-(PDMS)n-OAc was successfully obtained from HO-(PDMS)n-OH and acryloyl chloride (Figure 1a). The completeness of acrylation was confirmed by peaks of acrylic (C=C) and ester linkage (carbonyl group) bonds at 1633 cm^−1^ and 1708 cm^−1^, respectively, in the FTIR spectra of the product given in Figure 1b [31].

**Figure 2:**
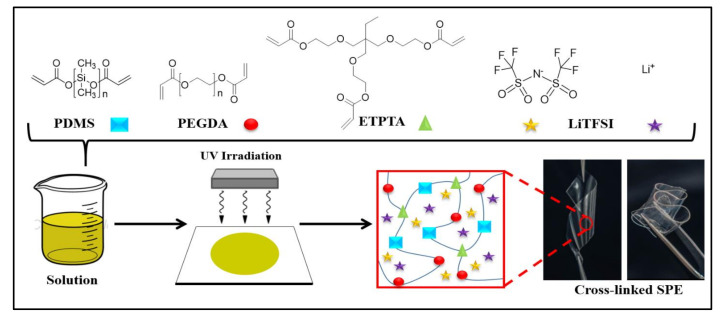
Sketched illustration of crosslinked PPE SPE film preparation, and representative photograph of a free-standing flexible PPE10.

Furthermore, crosslinked flexible polymer films were prepared by UV-irradiation of the polymer mixture, composed of a-PDMS, PEGDA, and ETPTA with LiTFSI. The sketched representation of the crosslinked PPE SPE film is shown in Figure 2, and the formulations of the PPE SPE films are given in Table 1. All thin polymer films were transparent, freestanding, and highly flexible. The photograph in Figure 2 shows a SPE, which is a free-standing PPE10 film with a maximum Li salt concentration ([EO]/[Li+] = 6). The completeness the of UV-crosslinking reaction of the PPE films was confirmed by monitoring the presence of the FTIR peaks of acrylic C=C bonds (1610–1625 cm^−1^) of the ETPTA monomer, AcO-(PDMS)n-OAc, and PEGDA before and after UV-irradiation. 

**Figure 3:**
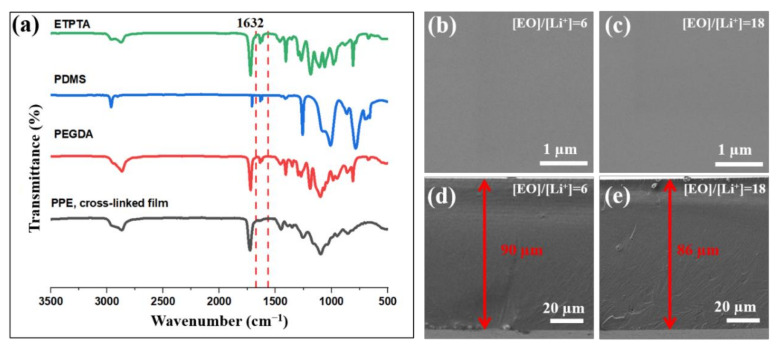
(**a**) FTIR spectra of crosslinked PPE film, and a representative (**b**,**c**) SEM, (**d**,**e**) cross-section images of flexible PPE10 SPE with different Li salt loadings.

As seen from Figure 3a, after the UV-photopolymerization process the peak of the acrylic bonds disappeared, which confirmed that the cross-linked PPE films were successfully formed. Additionally, the PPE films were characterized by measuring their gel fraction by solvent (acetone) extraction. It should be noted that for all the prepared PPE formulations, the gel fraction was found to be higher than 99%. This indicates that all obtained films were highly crosslinked. As seen from the SEM images in Figure 3b,c, the surface of the PPE SPEs is smooth and homogenous (without any agglomerated salt particles) which proves the complete dissociation of Li salt within the polymer. The absence of pores and wrinkles on the surface of the SPEs indicates the uniform morphology of a crosslinked network film, without phase separation or aggregation of the components. The thickness of the films was in a range from 90 to 150 µm (Figure 3d,e). The results of SEM/EDS analysis, showing the elemental mapping of C, S, F, and Si, also confirmed the homogenous distribution of a-PDMS and Li salt in SPEs (Figure S1).

**Figure 4:**
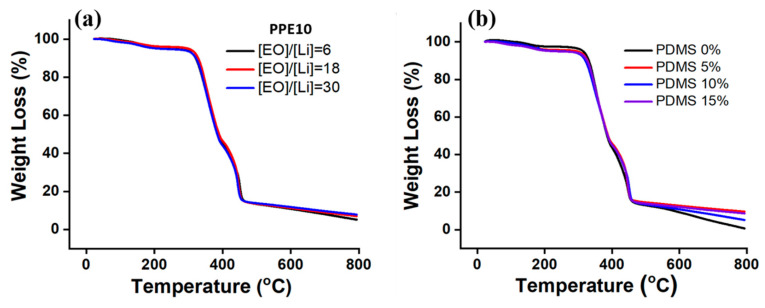
Thermo-gravimetric analysis of SPEs with various amounts of (**a**) LiTFSI and (**b**) a-PDMS ([EO]/[Li^+^] = 6).

The thermal behavior of the samples was examined by thermal gravimetric analysis (TGA), and thermogram profiles are given in Figure 4. The slight weight loss in all samples of about 3–4% below 150 °C was due to the moisture absorbed during the preparation and handling of the samples (Figure 4a,b). The thermal degradation of all samples started from 300 °C, which corresponds to the decomposition of polymers and LiTFSI [37]. It was observed from the thermogram that the increase of Li salt amount did not significantly affect the thermal stability of the PPE SPEs as seen from Figure 4a. Both the T_10_ (the temperature of 10 wt % weight loss) and T_50_ (50% weight loss) values of the films with different Li salt amounts and a-PDMS loading were very similar (Figure 4b). The values of T_10_ and T_50_ were observed as about 320 °C and 380 °C, respectively. These results indicate that the PPE membrane can be safely applied as a SPE at high temperature ranges.

The mechanical properties of the crosslinked polymer films were evaluated by standard tensile stress–strain tests, and the results are summarized in Table 1. There is no significant change in Young’s modulus value of the SPEs observed by variation of a-PDMS amount (0−15 wt %). However, it decreased with the increase of Li salt amount, which was expected due to the incorporation of salt with the polymer chains. The presence of salt increases the flexibility of polymer chains, and leads to a weakening of the mechanical strength [38]. However, by increasing the a-PDMS content, the tensile strength slightly decreased and the elongation at break increased, which was expected due to the chain flexibility of the polysiloxanes [22]. Nevertheless, the mechanical strength of the all thin films was high enough to act as a freestanding solid electrolyte in lithium-ion batteries, preventing short-circuit and enabling safe performance.

**Figure 5:**
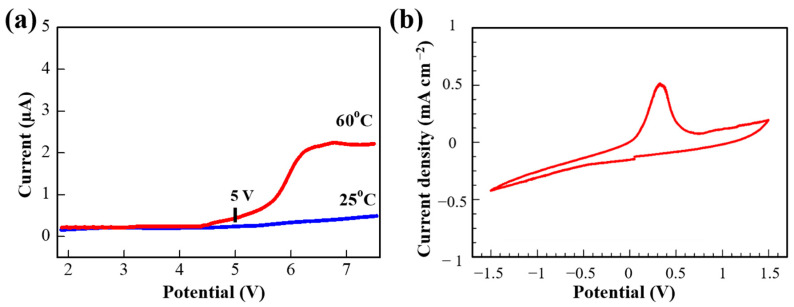
(**a**) Linear sweep voltammogram (LSV) and (**b**) cyclic voltammetry (CV) of SS/PPE10/Li coin cell with SPE PPE10 ([EO]/[Li^+^] = 6) at 60 °C.

The electrochemical stability window is an important parameter of SPEs for application in LIBs. Therefore, LSV measurements were conducted using a stainless steel/SPE/Li coin cell configuration at 25 and 60 °C. As shown in Figure 5a, the oxidative degradation of the PPE10 SPE film was not observed at room temperature, and starts to rise to about 5 V at 60 °C (Figure 5a,b). Additionally, the electrochemical stability window of the SPEs was tested by assembling blocking cells with stainless steel (SS), SS|SPE|SS (Figure S2). Both assembled cells showed a similar LSV pattern, and the potential window of the crosslinked PPE SPEs is large enough to be applied for high-voltage lithium-ion batteries. The stability window of PPE10 was significantly wider than that of the conventional linear PEO-based electrolyte, which is electrochemically unstable above 3 V [32,39]. Figure 5b shows the CV profile of the stainless steel/SPE/Li cell in the range of −1.5 to 1.5 V at a scan rate of 0.1 mV s^−1^. It depicts the reversible lithium plating and stripping processes. In the negative scan, the rising current was observed due to the lithium plating on the stainless steel electrode. The lithium stripping current peak was observed at about 0.4 V vs. Li/Li^+^. This confirms the effective operation of the SPE in the cell providing lithium-ion transportation.

**Figure 6:**
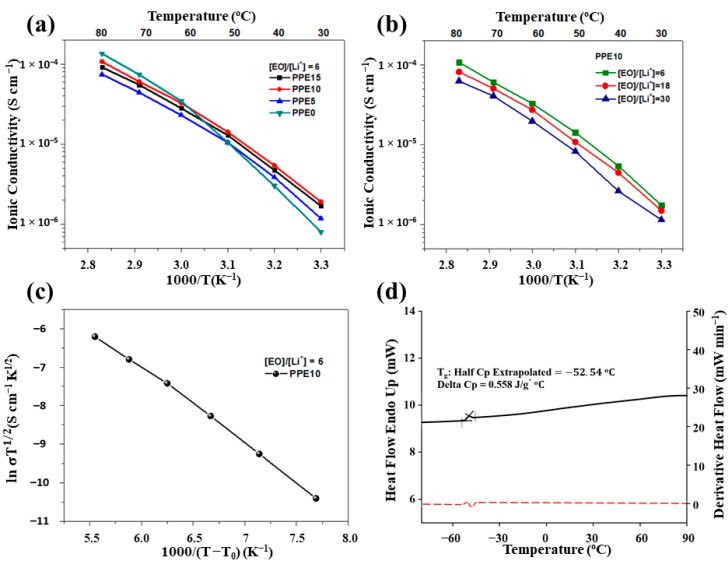
Ionic conductivity of the PPE SPEs with various amounts of (**a**) a-PDMS and (**b**) Li salt, as a function of temperature plots, (**c**) Vogel–Tamman–Fulcher fitting curve for PPE10, and (**d**) differential scanning calorimetry (DSC) profile for PPE15 solid polymer electrolytes.

The ionic conductivity of PPE SPEs sandwiched between two stainless steel discs (SS|PPE|SS) was measured by EIS within a temperature range from 30 °C to 80 °C. The temperature-dependent ionic conductivity of the samples, with different amount of a-PDMS and Li salt ([EO]/[Li^+^] = 6 is given as representative), were investigated, and the results are shown in Figure 6a,b. The conductivity increased with an increase of a-PDMS concentration. It is important to point out that the presence of a-PDMS significantly improved the ionic conductivity of all samples at lower temperature. An attempt to increase the a-PDMS concentration above 15 wt % led to the phase separation of the polymer mixture, possibly due to the hydrophobic nature of polysiloxanes. The ion conductivity of PPE10 film with various [EO]/[Li^+^] ratio is presented in Figure 6b. The increase of Li salt amount and its dissociation resulted in a higher amount of lithium ions, leading to higher ionic conductivity. The dissociation of LiTFSI occurs due to the strong interaction of lithium cations with the EO groups of both PEGDA and ETPTA. Therefore, the PPE SPEs can maintain a high level of conductivity at extremely high lithium salt concentrations. SPE PPE10, with a high salt amount ([EO]/[Li^+^] = 6) was able to show ionic conductivity of 1.75 × 10^−6^ S cm^−1^ at room temperature, and 1.07 × 10^−4^ S cm^−1^ at 80 °C. The further increase of salt concentration in such systems is limited by the poor solubility of Li salt in a polymer mixture, and the decrease of mechanical properties of freestanding polymer films. Despite the high content of Li salt, all SPEs with [EO]/[Li^+^] = 6 exhibited excellent mechanical properties and flexibility, without any supporting backbone, due to their crosslinked structure and the presence of polar functional groups. The a-PDMS has a low barrier energy to bond rotation and flexible polymer chains with low T_g_, which positively affect the lithium-ion conduction in a solid electrolyte system [22].

Moreover, PEGDA and ETPTA make easier the transport of Li+ ions in the polymer network, due to the pendant ethoxy groups in the structure [31]. Both of them affect the crosslinking density of the final networks, thus tailoring the physical properties of the cured films. A low T_g_ value is a key factor for achieving high ionic conductivity of SPEs, because lithium ions in the polymer matrix are transferred by the segmental motion of the polymer chains [22]. In the studied system, a-PDMS increased the amorphousness of the polymer film, which was confirmed by the DSC profile, and enabled Li^+^ ion movement. There were no phase transition points observed, and the presence of only glass transitions in the DSC profiles indicated the absence of crystalline phases [40]. The crystallinity of PEGDA was disrupted, since its chain ends are covalently linked (randomly) to a-PDMS, and ETPTA (Figure 6d). All DSC profiles of the samples showed very similar patterns, and T_g_ values are given in Table 1. As was expected, the increase of a-PDMS content decreased the T_g_ of the polymer films, hindering crystallization of the film. On the contrary, a rise of Li salt content slightly increased the T_g_ of SPEs, which can be attributed to the additional physical crosslinks of lithium ions between polymer chains, decreasing the molecular mobility of the network polymer chains [31,41]. Notably, the cross-linked network polymer maintained a relatively high ionic conductivity at lower temperature compared to the conventional PEO-based electrolytes. The conductivity value for PPE10 was nearly two orders of magnitude larger than that of conventional high molecular weight PEO homopolymers [32]. This was mainly due to the prevention of crystallization in the cross-linked structure, and the large chain motion range associated with flexible chains of a-PDMS in the system [22]. Additionally, the experimental data of PPE10 were considered by the VTF equation in order to determine a conductivity mechanism. As can be seen from the plot $ln\;\sigma T^{1/2}$ vs. 1000/(T−T_0_), the transportation of Li ions mostly occurred with the segmental motion of the polymer chains, which is expressed as a linear increment by temperature [35]. Compared with the reviewed linear polysiloxanes-based electrolytes by Wang et al., cross-linked PPE SPEs electrolytes exhibit superior dimensional stabilities and mechanical strength, even at high temperatures [42]. Moreover, PPE10 SPEs exhibits better ionic conductivity, and demonstrates stable Li plating/stripping processes and cycling stability toward metallic Li, as well as better flexibility, compared to similar cross-linked polysiloxane-based solid polymer electrolytes [24,26,29].

**Figure 7:**
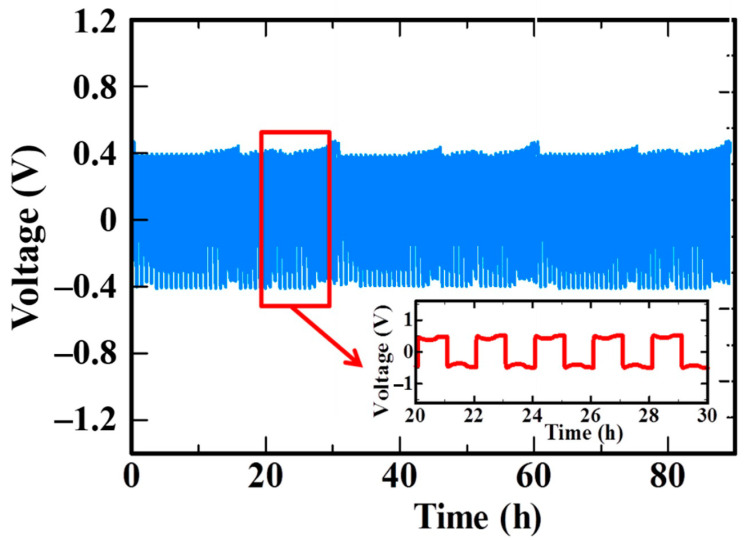
Galvanostatic cycling voltage profiles of SPE PPE10 ([EO]/[Li^+^] = 6) in a Li/SPE/Li symmetrical coin cell with a current density of 0.2 mA/cm^2^ at 60 °C. Magnification of selected cycles is shown in the inset.

In order to investigate the compatibility of SPE with the Li metal anode, the SPE PPE10 ([EO]/[Li^+^] = 6) was sandwiched in the Li–Li symmetrical cell and charged/discharged at 60 °C for 30 min under a current density of 0.2 mA cm^−2^ for each step. The time-dependent voltage profile of the symmetric cell shows that the voltage of the Li/SPE/Li cell could be stabilized at 400 mV, at a current density of 0.2 mA cm^−2^ (Figure 7). The positive and the negative voltages are relevant to the lithium stripping and plating processes, respectively. No short circuit feature was observed, and the voltage remained almost constant for over 90 h, confirming a stable SPE/Li interphase and excellent dielectric properties. The high compatibility of polysilaxane-based SPEs with lithium metal anodes was previously reported [42]. This characteristic enables the SPE to be assembled for high energy-density lithium metal batteries. 

## 4. Conclusions

In summary, a series of UV-crosslinked flexible SPE films with a-PDMS were developed and studied. Among the prepared SPEs, PPE10 had an ionic conductivity of 1.75 × 10^−6^ S cm^−1^ at room temperature, and 1.07 × 10^−4^ S cm^−1^ at 80 °C. The PDMS containing SPEs have improved ionic conductivity at lower temperature compared to conventional PEO based SPEs, by two orders of magnitude. The excellent mechanical strength and flexibility of the developed SPEs allows fabricating thin films of less than 100 µm in thickness, ensuring high ionic conduction and safe performance of LIBs. In addition, the developed crosslinked a-PDMS-based PPE SPEs have high electrochemical stability, which enables their application in high-voltage lithium ion batteries. The results of both the TGA and DSC profiles revealed that crosslinked SPE films with a-PDMS are thermally stable, have no phase changes, and could be safely applied at higher temperature as an alternative electrolyte. Another attractive feature is that, through Li/SPE/Li systematic cell investigations, SPE PPE10 ([EO]/[Li^+^] = 6) demonstrated superior compatibility toward metallic lithium. Thus, PPE SPEs are promising candidates for next-generation all-solid-state LIBs with high energy density. The future perspectives on PPE SPEs advancement include the development of flexible, high energy density batteries, by in situ synthesis of SPE at the surface of a high voltage cathode.

## Figures and Tables

**Table 1:** The different compositions of UV-photocrosslinked PPE solid polymer electrolytes and their mechanical properties.

	(a-PDMS:PEGDA:ETPTA) (wt %)	[EO]/[Li^+^]	T_g_ (°C)	Tensile Strength (MPa)	Elongation at Break (%)	Young’s Modulus (MPa)
**PPE0**	0:80:20	6	−44	1.68	22.99	14.03
**PPE5**	5:75:20	6	−49	1.41	27.50	16.61
**PPE10**	10:70:20	6	−50	1.45	26.6	16.03
**PPE15**	15:65:20	6	−53	1.30	28.96	14.22
**PPE10**	10:70:20	18	−51	1.66	24.52	20.75
**PPE10**	10:70:20	30	−55	1.72	25.37	34.75

## Supplementary Materials

The following are available online at https://www.mdpi.com/2073-4360/13/1/15/s1, Figure S1: SEM/EDS mapping showing the distribution of carbon (C), sulfur (S), fluorine (F) and silicon (Si) in PPE10 SPE with [EO]/[Li^+^] = 6., Figure S2: LSV of SS/PPE10/SS coin cell with SPE PPE10 ([EO]/[Li^+^] = 6) at 60 °C.

## References

[1] Manthiram A. (2017). An Outlook on Lithium Ion Battery Technology. ACS. Cent. Sci..

[2] Mentbayeva A., Sukhishvili S., Naizakarayev M., Batyrgali N., Seitzhan Z., Bakenov Z. (2020). Ultrathin clay-containing layer-by-layer separator coating enhances performance of lithium-sulfur batteries. Electrochim. Acta.

[3] Kalybekkyzy S., Mentbayeva A., Yerkinbekova Y., Baikalov N., Kahraman M.V., Bakenov Z. (2020). Electrospun 3D Structured Carbon Current Collector for Li/S Batteries. Nanomaterials.

[4] Kalybekkyzy S., Mentbayeva A., Kahraman M.V., Zhang Y., Bakenov Z. (2019). Flexible S/DPAN/KB Nanofiber Composite as Binder-Free Cathodes for Li-S Batteries. J. Electrochem. Soc..

[5] Wang J., Zhang L., Zhou Q., Wu W., Zhu C., Liu Z., Chang S., Pu J., Zhang H. (2017). Ultra-flexible lithium ion batteries fabricated by electrodeposition and solvothermal synthesis. Electrochim. Acta.

[6] Zhu M., Wu J., Wang Y., Song M., Long L., Siyal S.H., Yang X., Sui G. (2019). Recent advances in gel polymer electrolyte for high-performance lithium batteries. J. Energy Chem..

[7] Tolganbek N., Mentbayeva A., Uzakbaiuly B., Kanamura K., Bakenov Z. (2020). Li1+xAlxTi2−x (PO4)3, NASICON-type solid electrolyte fabrication with different methods. Mater. Today Proc..

[8] Fu G., Soucek M.D., Kyu T. (2018). Fully flexible lithium ion battery based on a flame retardant, solid-state polymer electrolyte membrane. Solid State Ion..

[9] Aziz S.B., Woo T.J., Kadir M.F.Z., Ahmed H.M. (2018). A conceptual review on polymer electrolytes and ion transport models. J. Sci. Adv. Mater. Devices.

[10] MacGlashan G.S., Andreev Y.G., Bruce P.G. (1999). Structure of the polymer electrolyte poly(ethylene oxide)6:LiAsF6. Nature.

[11] Chen R., Qu W., Guo X., Li L., Wu F. (2016). The pursuit of solid-state electrolytes for lithium batteries: From comprehensive insight to emerging horizons. Mater. Horiz..

[12] Meyer W.H. (1998). Polymer Electrolytes for Lithium-Ion Batteries. Adv. Mater..

[13] Fenton D.E., Parker J.M., Wright P.V. (1973). Complexes of alkali metal ions with poly(ethylene oxide). Polymer.

[14] Armand M. (1994). The history of polymer electrolytes. Solid State Ion..

[15] Arya A., Sharma A.L. (2017). Polymer electrolytes for lithium ion batteries: A critical study. Ionics.

[16] Agrawal R.C., Pandey G.P. (2008). Solid polymer electrolytes: Materials designing and all-solid-state battery applications: An overview. J. Phys. D Appl. Phys..

[17] Zhou D., Shanmukaraj D., Tkacheva A., Armand M., Wang G. (2019). Polymer Electrolytes for Lithium-Based Batteries: Advances and Prospects. Chem.

[18] Boaretto N., Joost C., Seyfried M., Vezzù K., Di Noto V. (2016). Conductivity and properties of polysiloxane-polyether cluster-LiTFSI networks as hybrid polymer electrolytes. J. Power Sources.

[19] Wang H., Cui X., Zhang C., Gao H., Du W., Chen Y. (2020). Promotion of Ionic Conductivity of PEO-Based Solid Electrolyte Using Ultrasonic Vibration. Polymers.

[20] Rossi N.A.A., Zhang Z., Schneider Y., Morcom K., Lyons L.J., Wang Q., Amine K., West R. (2006). Synthesis and characterization of tetra- and trisiloxane-containing oligo(ethylene glycol)s- highly conducting electrolytes for lithium batteries. Chem. Mater..

[21] Zhang Z., Jin J., Bautista F., Lyons L., Shariatzaden N., Sherlock D., Amine K., West R. (2004). Ion conductive characteristics of cross-linked network polysiloxane-based solid polymer electrolytes. Solid State Ion..

[22] Shim J., Kim L., Kim H.J., Jeong D., Lee J.H., Lee J.C. (2017). All-solid-state lithium metal battery with solid polymer electrolytes based on polysiloxane crosslinked by modified natural gallic acid. Polymer.

[23] Sutton P., Airoldi M., Porcarelli L., Olmedo-Martínez J.L., Mugemana C., Bruns N., Mecerreyes D., Steiner U., Gunkel I. (2020). Tuning the Properties of a UV-Polymerized, Cross-Linked Solid Polymer Electrolyte for Lithium Batteries. Polymers.

[24] Deka J.R., Saikia D., Lou G.W., Lin C.H., Fang J., Yang Y.C., Kao H.M. (2019). Design, synthesis and characterization of polysiloxane and polyetherdiamine based comb-shaped hybrid solid polymer electrolytes for applications in electrochemical devices. Mater. Res. Bull..

[25] Nair J., Destro M., Gerbaldi C., Bella F. (2015). Siloxane Diacrylate-based All-Solid Polymer Electrolytes for Lithium Batteries. Int. J. Membr. Sci. Technol..

[26] Oh B., Vissers D., Zhang Z., West R., Tsukamoto H., Amine K. (2003). New interpenetrating network type poly(siloxane-g-ethylene oxide) polymer electrolyte for lithium battery. J. Power Sources.

[27] Chen L., Fan L. (2018). Dendrite-free Li metal deposition in all-solid-state lithium sulfur batteries with polymer-in-salt polysiloxane electrolyte. Energy Storage Mater..

[28] Boaretto N., Horn T., Popall M., Sextl G. (2017). Optimization of the transport and mechanical properties of polysiloxane/polyether hybrid polymer electrolytes. Electrochim. Acta.

[29] Grewal M.S., Tanaka M., Kawakami H. (2019). Free-standing polydimethylsiloxane-based cross-linked network solid polymer electrolytes for future lithium ion battery applications. Electrochim. Acta.

[30] Aytan E., Uğur M.H., Kayaman-Apohan N. (2017). Facile fabrication and characterization of polyimide nanofiber reinforced photocured hybrid electrolyte for Li-ion batteries. Polym. Adv. Technol..

[31] Nair J.R., Gerbaldi C., Destro M., Bongiovanni R., Penazzi N. (2011). Methacrylic-based solid polymer electrolyte membranes for lithium-based batteries by a rapid UV-curing process. React. Funct. Polym..

[32] Kwon S.J., Kim D.G., Shim J., Lee J.H., Baik J.H., Lee J.C. (2014). Preparation of organic/inorganic hybrid semi-interpenetrating network polymer electrolytes based on poly(ethylene oxide-co-ethylene carbonate) for all-solid-state lithium batteries at elevated temperatures. Polymer.

[33] Schoener C.A., Weyand C.B., Murthy R., Grunlan M.A. (2010). Shape memory polymers with silicon-containing segments. J. Mater. Chem..

[34] Dose M.E., Bosch P., Freeman B.D., Mcgrath J.E., Ri J.S., Lozano A.E. (2017). Crosslinking via UV irradiation to improve gas separation performance. RSC Adv..

[35] Puthirath A., Patra S., Pal S., Puthirath Balan A., Tharangattu N.N. (2017). Transparent flexible lithium ion conducting solid polymer electrolyte. J. Mater. Chem. A.

[36] Wang W., Alexandridis P. (2016). Composite Polymer Electrolytes: Nanoparticles Affect Structure and Properties. Polymers.

[37] Kerner M., Plylahan N., Scheers J., Johansson P. (2015). Ionic liquid based lithium battery electrolytes: Fundamental benefits of utilising both TFSI and FSI anions?. Phys. Chem. Chem. Phys..

[38] Toker Öztürk R.D., Uğur M.H., Gungor A., Kayaman-Apohan N. (2018). Naphthalene-based polyimide electrolytes for lithium-ion battery applications. Adv. Polym. Technol..

[39] Armand M.B., Duclot M.J., Rigaud P. (1981). Polymer solid electrolytes: Stability domain. Solid State Ion..

[40] Hsu S.T., Tran B.T., Subramani R., Nguyen H.T.T., Rajamani A., Lee M.Y., Hou S.S., Lee Y.L., Teng H. (2020). Free-standing polymer electrolyte for all-solid-state lithium batteries operated at room temperature. J. Power Sources.

[41] Piana G., Bella F., Geobaldo F., Meligrana G., Gerbaldi C. (2019). PEO/LAGP hybrid solid polymer electrolytes for ambient temperature lithium batteries by solvent-free, “one pot” preparation. J. Energy Storage.

[42] Wang Q., Zhang H., Cui Z., Zhou Q., Shangguan X., Tian S., Zhou X., Cui G. (2019). Siloxane-based polymer electrolytes for solid-state lithium batteries. Energy Storage Mater..

