# Supplementary material for: Fabrication of UV-Crosslinked Flexible Solid Polymer Electrolyte with PDMS for Li-Ion Batteries
Source: Polymers (Basel). 2020 Dec 23;13(1):15. doi: 10.3390/polym13010015 (PMC7793537; doi:10.3390/polym13010015)
Supplement: Supplementary file 1 [file polymers-13-00015-s001.pdf]

Supplementary Information

Fabrication of UV-Crosslinked Flexible Solid Polymer Electrolyte with PDMS for Li-ion Batteries

Sandugash Kalybekkyzy [1,2,3], Al-Farabi Kopzhassar [1], Memet Vezir Kahraman [3], Almagul Mentbayeva [1,2,*] and Zhumabay Bakenov, [1,2*]

[1] Department of Chemical and Materials Engineering, School of Engineering and Digital Sciences, National Laboratory Astana, Nazarbayev University, Nur-Sultan 010000, Kazakhstan; sandugash.kalybekkyzy@nu.edu.kz (S.K.); alfarabi.kopzhasar@gmail.com (A.-F.K.)

[2] Institute of Batteries LLC, Nur-Sultan, 010000 Kazakhstan

[3] Department of Chemistry, Marmara University, Istanbul 34722, Turkey; mvezir@marmara.edu.tr (M.V.K.)

* Correspondence: almagul.mentbayeva@nu.edu.kz (A.M.); zbakenov@nu.edu.kz (Z.B.); Tel.: +7-777-395-4749 (A.M.); +7-705-265-53-10 (Z.B.)

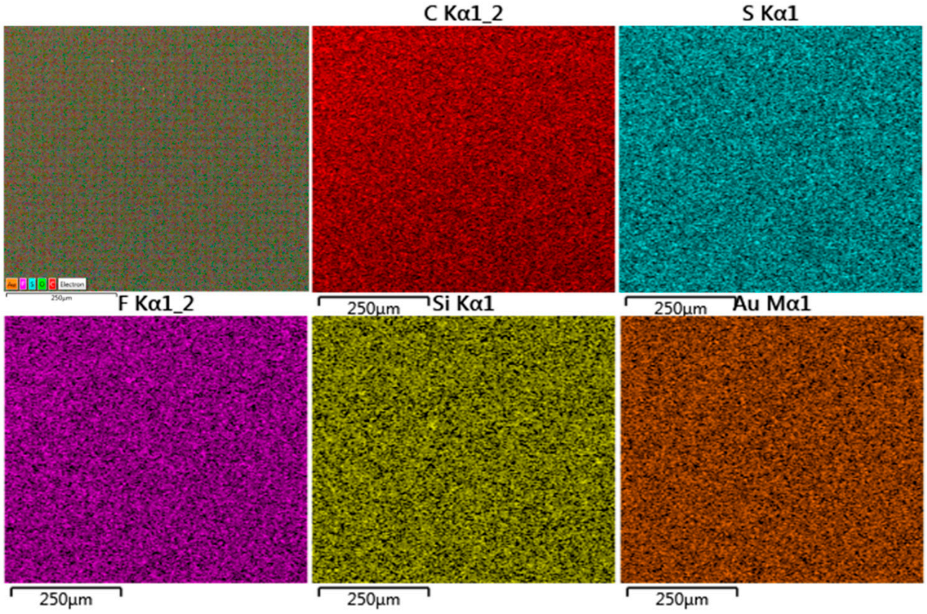

Figure S1. SEM/EDS mapping showing the distribution of carbon (C), sulfur (S), fluorine (F) and silicon (Si) in PPE10 SPE with $[EO]/[Li^+] = 6$.

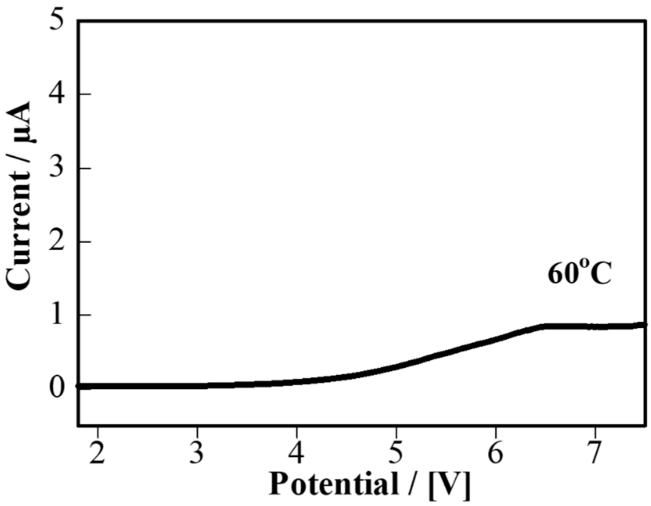

Figure S2. LSV of SS/PPE10/SS coin cell with SPE PPE10 ([EO]/[Li^+] = 6) at 60 °C.
